# The importance of waiting

**DOI:** 10.7554/eLife.03754

**Published:** 2014-07-22

**Authors:** Jarred Sanders, David Biron

**Affiliations:** 1 **Jarred Sanders** is in the Committee on Genetics, Genomics, and Systems Biology, The University of Chicago, Chicago, United States; 2 **David Biron** is in the Department of Physics, the James Franck Institute, and the Institute for Biophysical Dynamics, The University of Chicago, Chicago, United Statesdavid.biron@gmail.com

**Keywords:** G-CaMP, acetylcholine, ejaculation, dopamine, male mating behaviour, glutamate, *C. elegans*

## Abstract

Neural circuits that prevent a male *C. elegans* worm from copulating for several minutes after ejaculation have been identified.

**Related research article** LeBoeuf B, Correa P, Jee C, García LR. 2014. *Caenorhabditis elegans* male sensory-motor neurons and dopaminergic support cells couple ejaculation and post-ejaculatory behaviors. *eLife* **3**:e02938. doi: 10.7554/eLife.02938

**Image** The ability of a male *C. elegans* to ejaculate is linked to the activity of cells (shown in white) in the spicules used to penetrate the vulva of a hermaphrodite

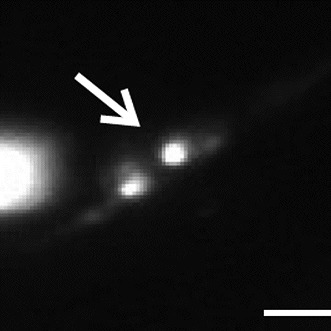

In his short story ‘Funes the Memorious’ Jorge Luis Borges tells of a young boy called Ireneo Funes who, after he was thrown off a wild horse, developed the ability to perceive everything and forget nothing. As a consequence, ‘he was not very capable of thought. To think is to forget a difference, to generalize, to abstract. In the overly replete world of Funes there were nothing but details, almost contiguous details’.

The ability to draw analogies between phenomena that are not identical, to identify imperfect patterns, is key to analytical thought and scientific discovery. Correspondingly, simple invertebrate model organisms have yielded some of the best insights into the cellular and molecular basis of behaviour in more complex organisms, including humans. Now, in *eLife*, René García and colleagues at Texas A&M University, including Brigitte LeBoeuf as first author, have closely examined one of the most complex behaviours exhibited by the roundworm *Caenorhabditis elegans*—male mating (LeBoeuf et al., 2014).

It may be surprising given their humble size, but male worms have a lot to do in very little time, as they are most sexually potent before the third day of adulthood (Guo et al., 2012). Given this time pressure, the drive to mate is likely one of the strongest motivators of a young adult male. Yet, despite the strength of this drive, males exhibit a period of sexual disinterest following ejaculation. While other species undergo a similar refractory period in response to sperm release, not much is known about the molecular and cellular mechanisms that control the downtime between mating events (Levin, 2009).

Mating is an intricate process in *C. elegans*. Most of the worms are hermaphrodites that can fertilize their own eggs, but there are also a small number of males that can fertilize the eggs in the hermaphrodites. The male responds to contact with a hermaphrodite by placing the copulatory apparatus in his tail flush with her body. He subsequently moves backwards—around her head or tail if necessary—until his tail contacts the vulva. The male then inserts two structures called spicules into the vulva that serve as mechanical anchors and also facilitate sperm transfer (Liu and Sternberg, 1995). Following penetration, sperm flows from where it is stored (the seminal vesicle) to a duct called the vas deferens in preparation for release, and after a few seconds it is ejaculated (Schindelman et al., 2006).

To identify a neural circuit that, as it turned out, coupled ejaculation to the refractory period, LeBoeuf et al. experimentally manipulated the refractory period. Cutting off the spicule tips reduced the time between subsequent matings. The spicules contain two sensory neurons, called the SPD and SPV neurons, with endings that are exposed inside the hermaphrodite uterus during copulation. Because males with severed spicules also had trouble ejaculating, LeBoeuf et al. wondered whether additional cells that regulate sperm transfer might control the time between matings. Since the neural circuit that controls sperm movement and release has been largely unexplored, they imaged the physiological activity of a group of male neurons and identified cells belonging to two categories: those involved in moving sperm to the vas deferens, and those that control sperm release (Figure 1). This circuit was also found to regulate the refractory period.

**Figure 1.**
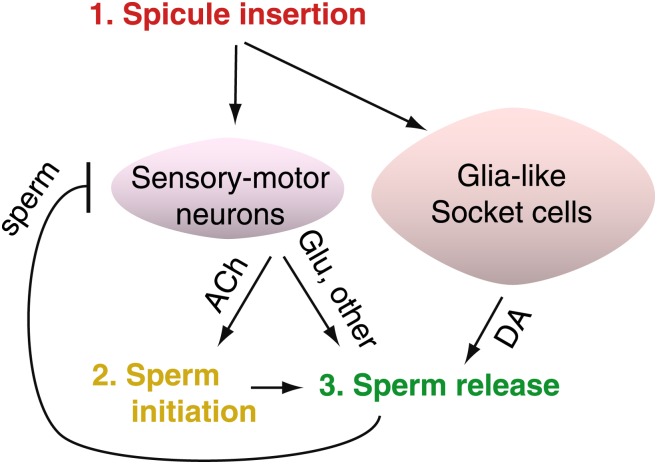
After *C. elegans* males ejaculate, sensory-motor neurons are inhibited, resulting in a period of reduced activity and mating ability. The spicules of a male *C. elegans* contain the sensory-motor neurons SPD and SPV. LeBoeuf et al. found that these SPD and SPV neurons stimulate cholinergic neurons to release acetylcholine (ACh) when the spicule is inserted into the hermaphrodite vulva. This begins the sperm initiation process (that is, sperm are moved from storage in the seminal vesicle to the vas deferens, ready for release). The sensory-motor neurons also stimulate a pair of glutamateric neurons called PCA to release glutamate (Glu), which triggers ejaculation. The release of sperm inhibits the activity of the sensory-motor neurons for several minutes after ejaculation; during this refractory period the male cannot copulate. LeBoeuf et al. also found that glia-like socket cells must also be present if proper ejaculation is to occur. These cells produce dopamine (DA), which controls sperm release and affects the length of the refractory period—more dopamine means a longer wait before ejaculation is possible again.

As expected, LeBoeuf et al. found that the SPD and SPV neurons were activated when the spicules entered the uterus. However, activity in these cells decreased only once sperm was released, suggesting that sensory cues from the hermaphrodite uterus affect this circuit. Additional experiments revealed that the SPD and SPV neurons relay information to the neurons that control the flow of sperm from the gonad and affect the ability of these neurons to prime the gonad for sperm movement. Moreover, the use of optogenetics—a technique in which light is used to control neurons—to stimulate ejaculation required activating many neurons that were not directly associated with the sex organs. Combining optogenetics with cell ablation—where cells are selectively destroyed—revealed one pair of glutamatergic sensory neurons (called PCA) with a particular role in sperm release. This is a classic example of circuit breaking in neuroscience.

In an unexpected and exciting twist, it was found that dopamine released from non-neuronal support cells called socket cells—which form a sheath around the sensory neurons—promotes ejaculation and increases the length of the refractory period. Cutting off the spicule tips removed both the spicule-associated sensory neurons and the socket cells, so damage to the socket cells could conceivably have impacted male mating. Destroying the socket cells reduced the males' ability to ejaculate and dopamine synthesis in these non-neuronal cells was necessary for sperm release. So does dopamine released from the socket cells also regulate the refractory period? Indeed, it does! Preventing the socket cells from synthesizing dopamine resulted in shorter refractory periods, and adding more dopamine extended them.

Understanding how small neural circuits underlie behaviour increasingly contributes to our thinking about more complex neural networks. This is the first study to identify a role for dopamine from neuronal support cells in the regulation of mating, and it also supports the idea that dopamine has an evolutionarily conserved role in promoting ejaculation (Peeters and Giuliano, 2008).

But why have a refractory period in the first place? One explanation is that this time is required for the neural circuitry to reset: for example, to remove the copious amounts of signalling molecules that flood the nervous system, or to desensitize receptors. Another—not mutually exclusive—possibility is that the refractory period could indirectly improve the fitness of the species by increasing the probability that the male will mate with multiple partners.

Uncovering the mechanisms that regulate the male mating drive, and how it might compete with other behavioural drives, is a developing story. The detailed understanding emerging about the roles of neuromodulation in shaping circuits and behaviour will be interesting to follow (Bargmann, 2012; Marder, 2012).

## References

[bib1] Bargmann CI 2012 Beyond the connectome: how neuromodulators shape neural circuits. Bioessays 34:458–465. doi: 10.1002/bies.201100185 22396302

[bib2] Guo X Navetta A Gualberto DG Garcia LR 2012 Behavioral decay in aging male *C. elegans* correlates with increased cell excitability. Neurobiology of Aging 33:e1485–1423. doi: 10.1016/j.neurobiolaging.2011.12.016 PMC3378242 22285759

[bib3] LeBoeuf B Correa P Jee C García LR 2014 *C. elegans* male sensory-motor neurons and dopaminergic support cells couple ejaculation and post-ejaculatory behaviors. eLife 3:e02938. doi: 10.7554/eLife.02938 24915976 PMC4103683

[bib4] Levin RJ 2009 Revisiting post-ejaculation refractory time-what we know and what we do not know in males and in females. The Journal of Sexual Medicine 6:2376–2389. doi: 10.1111/j.1743-6109.2009.01350.x 19515210

[bib5] Liu KS Sternberg PW 1995 Sensory regulation of male mating behavior in *Caenorhabditis elegans*. Neuron 14:79–89. doi: 10.1016/0896-6273(95)90242-2 7826644

[bib6] Marder E 2012 Neuromodulation of neuronal circuits: back to the future. Neuron 76:1–11. doi: 10.1016/j.neuron.2012.09.010 23040802 PMC3482119

[bib7] Peeters M Giuliano F 2008 Central neurophysiology and dopaminergic control of ejaculation. Neuroscience and Biobehavioral Reviews 32:438–453. doi: 10.1016/j.neubiorev.2007.07.013 17919726

[bib8] Schindelman G Whittaker AJ Thum JY Gharib S Sternberg PW 2006 Initiation of male sperm-transfer behavior in *Caenorhabditis elegans* requires input from the ventral nerve cord. BMC Biology 4:26. doi: 10.1186/1741-7007-4-26 16911797 PMC1564418

